# Semantic clustering analysis of E3-ubiquitin ligases in gastrointestinal tract defines genes ontology clusters with tissue expression patterns

**DOI:** 10.1186/s12876-022-02265-2

**Published:** 2022-04-12

**Authors:** Veronika Iatsiuk, Frantisek Malinka, Marketa Pickova, Jolana Tureckova, Jiri Klema, Frantisek Spoutil, Vendula Novosadova, Jan Prochazka, Radislav Sedlacek

**Affiliations:** 1 Laboratory of Transgenic Models of Diseases and Czech Centre for Phenogenomics, Institute of Molecular Genetics of the Czech Academy of Sciences, Prague, Czech Republic; 2 Department of Computer Science, Czech Technical University in Prague, Prague, Czech Republic

**Keywords:** Ub-ligase, GIT, Regeneration, Cluster analysis, Semantic biclustering, Gene redundancy

## Abstract

**Background:**

Ubiquitin ligases (Ub-ligases) are essential intracellular enzymes responsible for the regulation of proteome homeostasis, signaling pathway crosstalk, cell differentiation and stress responses. Individual Ub-ligases exhibit their unique functions based on the nature of their substrates. They create a complex regulatory network with alternative and feedback pathways to maintain cell homeostasis, being thus important players in many physiological and pathological conditions. However, the functional classification of Ub-ligases needs to be revised and extended.

**Methods:**

In the current study, we used a novel semantic biclustering technique for expression profiling of Ub-ligases and ubiquitination-related genes in the murine gastrointestinal tract (GIT). We accommodated a general framework of the algorithm for finding tissue-specific gene expression clusters in GIT. In order to test identified clusters in a biological system, we used a model of epithelial regeneration. For this purpose, a dextran sulfate sodium (DSS) mouse model, following with in situ hybridization, was used to expose genes with possible compensatory features. To determine cell-type specific distribution of Ub-ligases and ubiquitination-related genes, principal component analysis (PCA) and Uniform Manifold Approximation and Projection technique (UMAP) were used to analyze the Tabula Muris scRNA-seq data of murine colon followed by comparison with our clustering results.

**Results:**

Our established clustering protocol, that incorporates the semantic biclustering algorithm, demonstrated the potential to reveal interesting expression patterns. In this manner, we statistically defined gene clusters consisting of the same genes involved in distinct regulatory pathways vs distinct genes playing roles in functionally similar signaling pathways. This allowed us to uncover the potentially redundant features of GIT-specific Ub-ligases and ubiquitination-related genes. Testing the statistically obtained results on the mouse model showed that genes clustered to the same ontology group simultaneously alter their expression pattern after induced epithelial damage, illustrating their complementary role during tissue regeneration.

**Conclusions:**

An optimized semantic clustering protocol demonstrates the potential to reveal a readable and unique pattern in the expression profiling of GIT-specific Ub-ligases, exposing ontologically relevant gene clusters with potentially redundant features. This extends our knowledge of ontological relationships among Ub-ligases and ubiquitination-related genes, providing an alternative and more functional gene classification. In a similar way, semantic cluster analysis could be used for studding of other enzyme families, tissues and systems.

**Supplementary Information:**

The online version contains supplementary material available at 10.1186/s12876-022-02265-2.

## Background

Ubiquitination is the most common post-translational protein modification [1], during which a small protein, ubiquitin (Ub), is covalently attached to the substrate in a three-enzyme cascade reaction catalyzed by subsequent activation (E1), conjugating (E2) and ligation (E3) enzymes [2, 3]. Ubiquitination can either direct proteins for degradation, mediated by the proteasome system [3], or modulate their intracellular localization, vesicular trafficking [4], activation of signaling pathways and alteration of DNA transcription [2, 5]. Enzymes responsible for transferring ubiquitin to a given protein are E3 Ub-ligases [6]. According to the conserved domains and the mechanism of the Ub transfer to the substrate, E3 Ub-ligases have been divided into three basic classes: really interesting new genes (RINGs), homologous to the E6-AP C terminus (HECTs), and RING between RINGs (RBRs) [1, 7]. Individual Ub-ligases recognize their targets in a strictly regulated manner without any respect to their sequence similarities. To ensure high specificity during selection of target proteins, it has been predicted that more than 600 genes encode E3-Ub-ligases in the human genome [1], whilst there are only two E1 and 30–50 E2 genes, respectively [8]. Depicting the regulatory roles of Ub-ligases within complex regulatory networks can be hampered by strong parallel compensation mechanisms, Ub-ligases can often recognize the same substrate or affect different nodes of same regulatory pathway. This makes it difficult to predict alternative compensatory enzymes in reverse genetics approaches. For this reason, more functional classification of Ub-ligases is needed.

One of the ways E3 ubiquitin ligases are classified is according to function using their Gene ontology [9], which describes three aspects of the biological domain through molecular function, cellular component, and biological process in which it is involved [6]. Nowadays, one of the most popular methods that employs this type of classification is Gene Set Enrichment Analysis (GSEA) [10]. However, the ability of this method to describe complex biological phenomena is limited by the format of its input and output [11]. To improve this descriptive power, Semantic analysis method [12] and the sem1R algorithm [11] were introduced. These methods enable the determination and description of semantically comprehensive gene biclusters using a conjunction of ontological terms from various ontologies. Since the hypothesis language is thus extended, the method provides a more complete picture of functional gene classification for specific cell types in the tissue.

The gastrointestinal tract (GIT) is a system with a high rate of regeneration. It consists of a variety of diverse epithelial cell populations with varying morphology and function, such as nutrient absorption, hormone production, barrier function, responding to microorganisms, coordination of immune response, as well as self-renewal [13, 14]. In addition to a diverse population of epithelial cells, stem cells and mucosa-associated lymphoid tissue can be found along the GIT [15, 16]. Tissue specific stem cells of epithelial origin which continuously divide, proliferate and differentiate to ensure the turnover of cells and the overall tissue homeostasis [17]. There are multiple signaling pathways, such as Wnt, Notch, or EphrB3, which have been known to be critical for regulation of the stem cell niche and differentiation of progeny cells [14, 18]. These features are determined by unique gene signature and regulatory pathway cooperation that is individual to each specific cell type, and can be found in their RNA profile [19]. Therefore, GIT represents a valuable model system to study parallel regulatory networks in the context of tissue homeostasis, regeneration, and response during pathogenic processes.

However, little is known about how ubiquitin ligases are involved in such physiological regulatory processes, either in GIT or in other systems, despite increasing evidence that an aberrant function or dysregulation of the expression of the E3 Ub-ligases can cause pathological changes resulting in dysplasia, metaplasia or even cancer [20]. In this regard, understanding the function of individual Ub-ligases in tissue context would help to understand development of pathological conditions and eventually their therapeutic targeting [2, 21]. Thereby, in this study we aim to identify GIT specific Ub-ligases and ubiquitination-related genes, their role in tissue homeostasis and their possible contribution to alternative compensatory networks.

Here, we introduced a semantic clustering method [11, 12] combined with the expression profiles of E3 Ub-ligases and ubiquitination-related genes in the stomach, small intestine and colon, aiming to specify the dominant biological role of individual E3s, as well as potentially predict their secondary compensatory roles in different parts of GIT during tissue homeostasis and regeneration. In addition, by using already published single-cell RNA sequencing data [22], we attempt to identify cell-type specific Ub-ligases in the colon. We demonstrate that an individual Ub-ligase may be typical for several cell types, although its expression is determined by the current status of the tissue and could differ during injury response or regeneration.

## Methods

### Animals

For this study were used C57BL/6NCrl mice (Charles River Laboratories). For the expressional profiling, three 12-week-old C57BL/6NCrl males were used. Stomach, small intestine, and colon were dissected and immediately proceeded for RNA isolation.

### RNA isolation and reverse transcription

RNA was isolated with the TRI reagent (Sigma-Aldrich, USA) according to the manufacturer's recommendations. Parts of small intestine (duodenum, jejunum and ileum) or colon (proximal, distal) were pooled together into one sample from all animals. The appropriate RNA was transcribed into cDNA using GoScript™ Reverse kit (Promega, USA).

For expression profiling of Ub-ligase genes, kit RT2 Profiler PCR Arrays (Qiagen, USA) was used with different specific primer pair for each gene of E3-Ub ligase placed in each position that allows to measure 370 genes simultaneously. Chosen genes represent Ub-ligases and that could be potential drug targets. This array includes ubiquitin ligases from all major E3 families and important regulatory ubiquitination-related genes with suggested redundant or compensating functions that are not Ub-ligases by the nature, but could possibly cause lower drug efficacy or off-target effects. Internal controls for reference genes and detecting genomic DNA contamination were included within the plate: housekeeping genes (GAPDH, HSP90, beta-actin, Gus-B, beta-2 microglobulin), genomic DNA contamination control and positive PCR controls (see manufacturer's handbook).

### Mouse model of epithelial mucosa damage

Twelve-week-old C57BL/6NCrl males (Charles River Laboratories) were used for this study. For the epithelial damage studying the dextran sulfate sodium (DSS) mouse model was used [23]. Three males were treated with 2% DSS (w/v) (TdB Consultancy, Sweden) for five days. On the evening of the 5th day, DSS was exchanged for drinking water overnight to reach the peak phase of acute inflammation. Two animals were used as controls with plain drinking water only. On day 6, mice were sacrificed and intestinal tissues were processed for histology and RNA extraction. Stomach, small intestine and colon from sacrificed mice were dissected, fixed for 24 h in 10% buffered formaldehyde (v/v) (Thermo Scientific, USA) at 4 °C, embedded in paraffin and sectioned with automatic rotating microtome RM2255 (Leica Biosystems, Nussloch, Germany).

### RNA probe preparation and in situ hybridization

Primers for in situ hybridization were designed in Primer-BLAST (NCBI) covering at least one exon:exon border of the gene. The list of primers is shown in Additional file 3: Table S1. The DNA template was transcribed according to the probe sequence in plasmid (pGEM®-T Easy Vector Systems, Promega, USA) into mRNA probe by In vitro transcription with T7 or Sp6 RNA polymerase (Promega, USA) following the manufacturer’s protocol.

In situ hybridisation was performed on 7 μm paraffin tissue sections of the distal colon based on the protocol of Wilkinson, et al. [24]. Sections were deparaffinized, permeabilized with 10 µg/ml Proteinase K (Sigma-Aldrich, Germany), post fixed with 4% PFA and washed. Next, the sections were acetylated with acetic anhydride (Sigma-Aldrich, Germany) and washed. Slides were then treated with hybridization buffer for 1 h containing Formamide, 20 × SSC, pH 7.0 (Thermo Scientific, USA), 50 × Denhardt’s solution, 10% Tween-20, tRNA 10 mg/ml, Heparin 50 mg/ml and Salmon sperm DNA 10 mg/ml (all purchased from Sigma-Aldrich, Germany). Hybridization with specific anti-sense mRNA probes (2 ng/μl, denatured for 3 min at 80 °C) was done O/N in moisten chamber at 70 °C.

Thereafter, unspecific binding of mRNA was washed off the sections with 5xSSC-Formamide, pH 7.0 and then 2 × SSC, pH 7.0 (Thermo Scientific, USA) at 70 °C in water bath. Afterwards, slides were washed 4 times with TBS solution. The endogenous alkaline phosphatase was blocked with the Blocking reagent for 1 h (Roche, Switzerland). Digoxigenin-labelled mRNA probes were detected with anti-digoxigenin Fab fragments conjugated with alkaline phosphatase (Roche, Switzerland) at dilution 1 µl/5 ml of TBS, 4 °C O/N. The antibody was washed out with TBS. The visualization of signal was performed with BM-Purple solution (Roche, Switzerland). Post fixation was done with 4% buffered PFA and slides were mounted with Aquatex mounting medium (Merck Millipore, Germany).

### Statistical analysis

qPCR data were normalized on HSP90 gene expression. Missing data were replaced by maximum value + 2 for a given gene, recalculated to relative quantities and log transformed. The ANOVA test with Tukey post-test was used for analyzing different gene expression in different GIT parts. As significance level we used *p* = 0.01. Comparison of DSS treated and untreated distal colon was not performed due to small sample size. As primary criterion for selection potential interesting genes, the absolute difference higher than 1.25 delta Cq was used and all values from one had to be higher/smaller compared to any value from the second group. Fisher test was used for comparison of category data (distribution of ontology terms in different tissue and structural groups).

### Ontology analysis and semantic analysis

Ontologies that were used in all experiments are the following: Gene ontology [9], Pathway ontology [25], and KEGG Brite database [26]. These ontologies contain 45044, 2601, and 63263 ontological terms, respectively. We note that each term in ontology represents one biological knowledge and therefore the size and numbers of ontologies appended to the semantic analysis affect the ability to explain biological phenomena. In other words, ontologies with higher numbers of terms have a larger potential to describe a hypothesis (e.g. processes in genes) more precisely since their hypothesis language is more extensive. On the other hand, it has a negative impact on run time of algorithms. The ontological terms are arranged hierarchically, which means that one term might be more general then the others. For example, term “regulation of biological process” is more general than term “regulation of cellular process”. This hierarchical order might help to understand relations among the ontological terms and their biological meanings at different levels of specificity. To perform the semantic analysis and afterwards a semantic clustering for the specific part of GIT, the entire gene set was split into three groups—significant or not significant for each comparison (Small intestine vs colon—Group A, stomach vs colon Group B and stomach vs small intestine Group C). Then, the enrichment score (statistical significance) of each ontological term was calculated for each group of significant and non-significant differentially expressed genes, i.e. Group A, Group B, and Group C. For this analysis, *computeTermsEnrichment* function of sem1R algorithm [10] was used.

### Semantic cluster analysis

For semantic cluster analysis the sem1R algorithm induces a set of predictive rules that describe coherent biclusters using ontology terms from input data. In this case, the input data means a gene set of significant and non-significant genes for each comparison (Small intestine vs colon—Group A, stomach vs colon Group B and stomach vs small intestine Group C), and a set of ontologies. We note that the input data, i.e. groups of genes and ontologies, are the same that were used in the ontology and semantic analysis. Here, each rule was formulated as a conjunction of ontology terms, where a group of genes covered by the rule had to be associated with all ontology terms appearing in that rule. An example of such rule might be the following rule: cellular protein metabolic process ∧ protein phosphorylated amino acid binding.

The rule above defines a set of genes that are simultaneously associated with cellular protein metabolic process and with protein phosphorylated amino acid binding. A graphical representation of this rule is shown in Fig. 2D. Similar rules were used for computing Fig. 2B, F.

The concept of semantic cluster analysis is illustrated in Additional file 5: Fig. S1. The figure shows a process of inducing hypotheses for each set of significantly and non-significantly expressed genes of the original qPCR dataset that is divided into three groups of samples. Then, hypotheses in the form of a set of rules are induced using the sem1R algorithm.

### Selection rules definition

The selected groups of genes were sorted according to the t-score and number of differences between significantly and non-significantly expressed genes (minimum difference was set up arbitrarily equal to 3) for each ontology level. For each group (Group A, Group B, and Group C) we ran the sem1R algorithm that is restricted to find a maximum of 10 best rules (groups of genes) according to an evaluation function. To get more different rules and consequently more different covered groups of genes, all supported evaluation functions (ACC, AUC, and F1-score) were used in the process of rule learning. To control a level of specificity of rules, ‘minLevel’ parameter was set up to 0, 2, 3, 4, 5, and 6 for all runs of the sem1R algorithm. Defining a minimal level of specificity prevents to induce too general or too specific rules that cover too many or too few genes, respectively. From all of these runs of various settings, interesting rules and consequently corresponding groups of genes were selected.

## Results

### Expression profiling of E3 Ub-ligases demonstrates tissue-specific gene combinations in the gastrointestinal tract

**Fig. 1:**
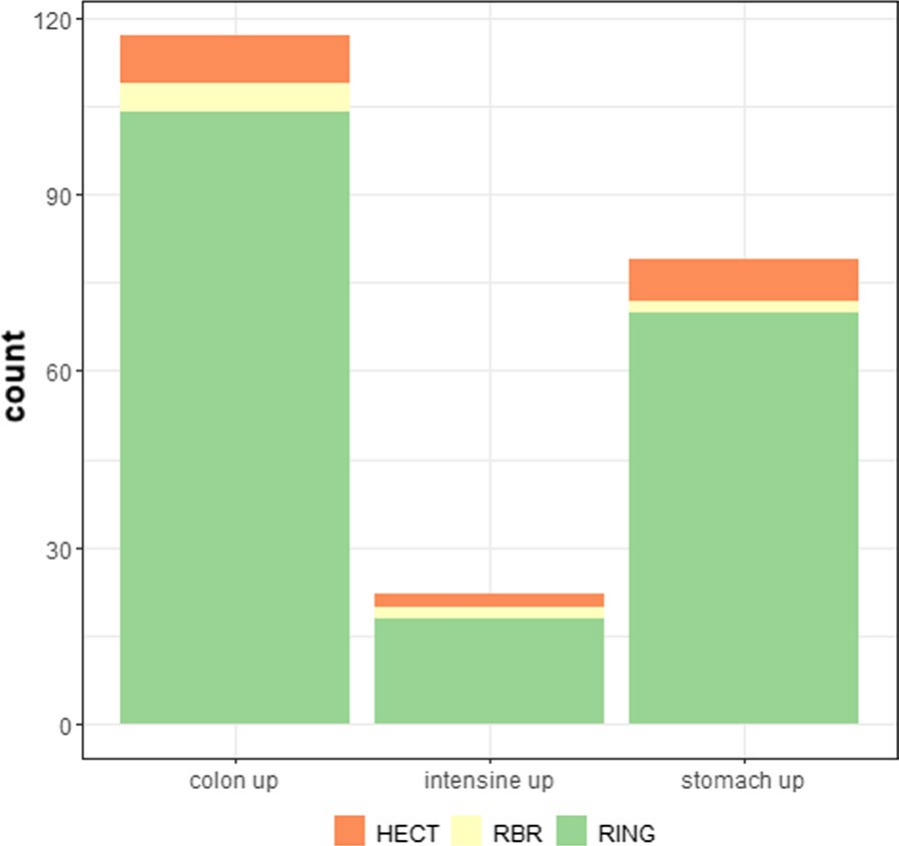
Representative distribution of upregulated genes in stomach, small intestine and colon divided into main Ub-ligase classes

The expression profiling of E3 Ub-ligases and ubiquitination-related genes was performed on samples from stomach, small intestine and colon of WT mice (Additional file 1). We found that each organ of GIT has their specific set of up- and downregulated genes (Fig. 1), suggesting their organ-specific roles. For further analysis, the genes were divided into three groups, corresponding to defined Ub-ligases types. In colon samples, we detected 118 upregulated genes (sum of upregulated genes compared to intestine or stomach), in intestine 22 were upregulated (sum of upregulated genes compared to colon or stomach) and in stomach there were 78 upregulated (sum of upregulated genes compared to colon or intestine). No significant difference was found in the representation of individual Ub-ligases types with a *p *value 0.736 for upregulated genes in each organ. Genes from this cluster were expressed at the same level in stomach, small intestine and colon and might have the same functional activity for each organ.

In the next step, we defined ontological terms for each gene and compared the distribution of ontology terms with theoretical distribution. We found 26 significantly enriched terms for genes, which were differentially expressed in specific parts of GIT (Table 1; Additional file 3: Table S2). These ontology terms displayed specific functions of given genes.

**Table 1:** Ontologies significantly different for different tissue types

Ontology function/activity place	*p* value	Colon up	Intestine up	Stomach up	Ontology function/activity place	*p* value	Colon up	Intestine up	Stomach up
Cellular response to cytokine stimulus	0.011	0.00%	9.09%	0.00%	Negative regulation of protein localization to nucleus	0.032	0.85%	9.09%	0.00%
			Socs1				Trim40	Trim40	
Cellular response to organic cyclic compound	0.032	0.85%	9.09%	0.00%	Negative regulation of signal transduction	0.02	0.85%	13.64%	2.56%
		Btrc	Socs1				Socs5	Socs1 Socs3	Cish Socs5
Cytokine-mediated signaling pathway	0.02	0.85%	13.64%	2.56%	Negative regulation of tyrosine phosphorylation of STAT protein	0.001	0.00%	13.64%	0.00%
		Socs5	Socs1 Socs3 Cish	Socs5				Socs1 Socs3	
Cytoplasmic ribonucleoprotein granule	0.011	0.00%	9.09%	0.00%	Positive regulation of I-kappaB kinase/NF-kappaB signaling	0.042	1.69%	13.64%	2.56%
			Socs1				Mul1 Trim62	Mul1 Rnf31 Trim30	Trim13 Trim62
IkappaB kinase complex	0.032	0.85%	9.09%	0.00%	Positive regulation of regulatory T cell differentiation	0.011	0.00%	9.09%	0.00%
		Trim40	Trim40					Socs1	
Insulin-like growth factor receptor binding	0.011	0.00%	9.09%	0.00%	Protein kinase inhibitor activity	0.02	0.85%	13.64%	2.56%
			Socs1				Socs5	Socs1 Socs3	Socs5 Cish
Intracellular signal transduction	0.049	3.39%	18.18%	5.13%	Protein neddylation	0.032	0.85%	9.09%	0.00%
		Socs5 Spsb4 Wsb1	Socs1 Socs3 Wsb1	Asb2 Cish Socs5			Trim40	Trim40	
JAK-STAT cascade	0.042	1.69%	13.64%	2.56%	Regulation of activation of Janus kinase activity	0.011	0.00%	9.09%	0.00%
		Pias1 Socs5	Socs1 Socs3	Pias1 Socs5				Socs1	
Negative regulation of insulin receptor signaling pathway	0.002	0.00%	13.64%	1.28%	Regulation of cytokine secretion	0.011	0.00%	9.09%	0.00%
			Socs1 Socs3	Cish				Socs1	
Negative regulation of JAK-STAT cascade	0.02	0.85%	13.64%	2.56%	Regulation of interferon-gamma-mediated signaling pathway	0.011	0.00%	9.09%	0.00%
		Socs5	Socs1 Socs3	Cish Socs5				Socs1	
Negative regulation of NF-kappaB transcription factor activity	0.032	0.85%	9.09%	0.00%	Regulation of JAK-STAT cascade	0.011	0.00%	9.09%	0.00%
		Trim40	Trim40					Socs1	
Negative regulation of protein catabolic process	0.032	0.85%	9.09%	0.00%	Regulation of protein phosphorylation	0.001	0.00%	13.64%	0.00%
		Trim40	Trim40					Socs1 Socs3	
Negative regulation of protein kinase activity	0.02	0.85%	13.64%	2.56%	Regulation of tyrosine phosphorylation of STAT protein	0.011	0.00%	9.09%	0.00%
		Socs5	Socs1 Socs3	Socs5 Cish				Socs1	

Ontology clusters of stomach represent genes that are involved in stress responses by regulating various intracellular signal transduction with association of the SCF ubiquitin ligase [27]. Ontology groups display that the small intestine is mostly represented by genes playing roles in immune and inflammatory responses. This ontology group is represented by the suppressor of cytokine signaling (SOCS) family of protein-encoded genes Socs1 and Socs3. These genes are responsible for negative regulation of cytokine signaling through the JAK/STAT3 pathway, and was mentioned as a probable substrate recognition component of a SCF-like ECS E3 ubiquitin-protein ligase complex [28, 29]. Next, there was a group of upregulated genes in the small intestine (14% out of all upregulated), which are responsible for negative regulation of the insulin receptor signaling pathway (Table 1; Additional file 2). The most representative gene for this group was Cish, which is also a member of SOCS family [30].

Ontology clustering the upregulated genes revealed enrichment of genes specifically involved in DNA repair, apoptosis, and catabolic processes. For instance, upregulation of the E3 ubiquitin-protein ligase Trim62 is a positive regulator of I-κB kinase/NF-κB signaling and DNA-binding of transcription factors (Table 1; Additional file 2) [31]. Mul1 and Trim13 (also known as Ret finger protein 2, RFP2), among the others were genes that take a role in positive regulation of cell death by modulating the innate immune response against viruses [32].

**Fig. 2:**
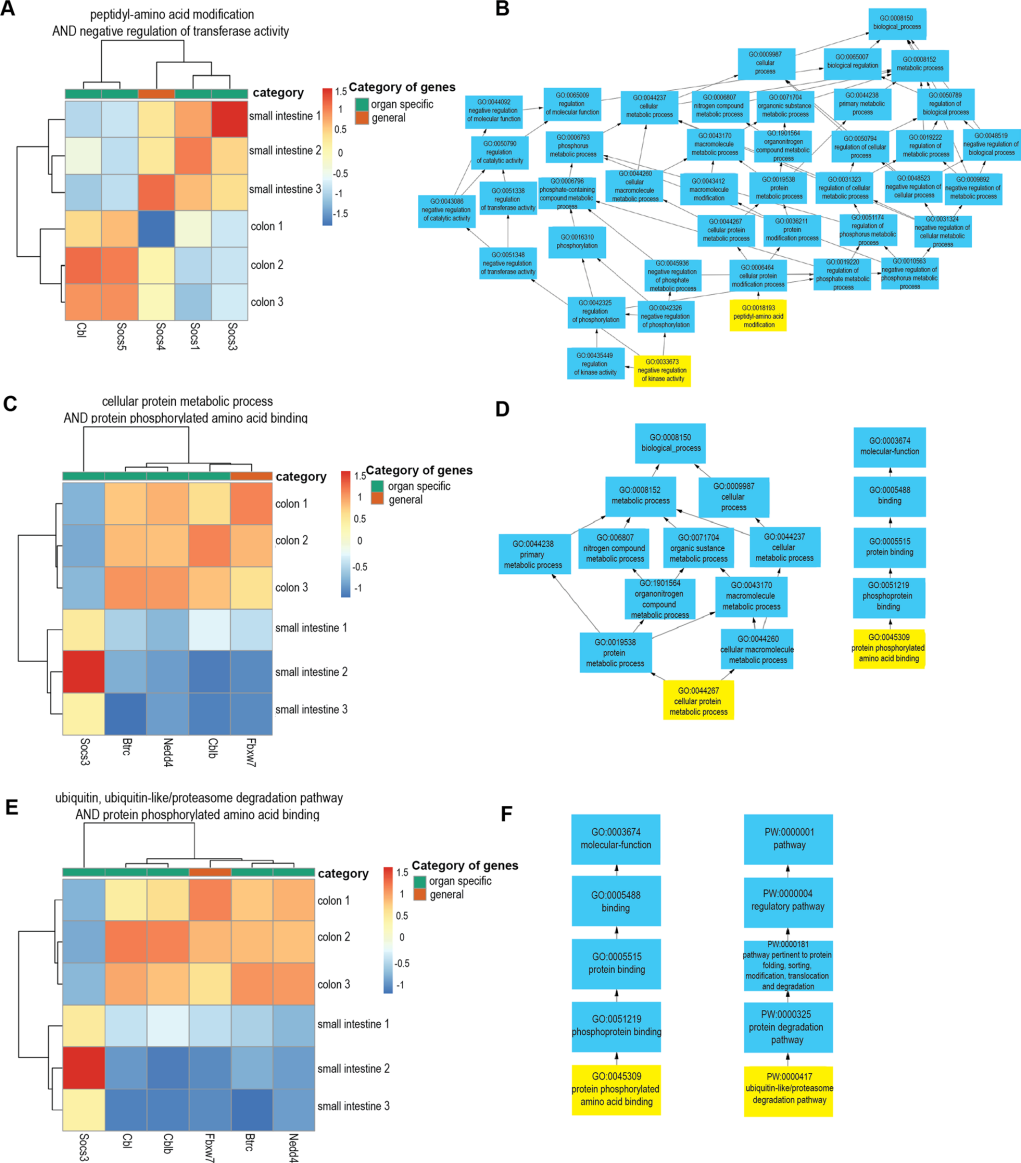
Selected examples of ontologically related genes in GIT. Heatmaps for organ specific (green columns, top) and general genes (orange columns, top) that are annotated simultaneously by ontology terms GO:0018193: peptidyl-amino acid modification and GO:0042326: negative regulation of phosphorylation (**a**), GO:0045309: protein phosphorylated amino acid binding and GO:0044267: cellular protein metabolic process (**c**), and GO:0045309: protein phosphorylated amino acid binding and PW:0000417: ubiquitin, ubiquitin like/proteasome degradation pathway (**e**). Schematic visualization of gene ontology and their more general terms for GO:0018193 and GO:0042326 (**b**), GO:0045309 and GO:0044267 (**d**), GO:0045309 and PW:0000417 (**f**). We note that these terms are highlighted by yellow color. Selected ontologies illustrate the ability of semantical clustering to group genes that carry the same biological function in different parts of the organ. Scheme shows the relationships among ontology terms related to biological processes that might help the analyst to orient in such extensive ontologies and then interpret the meaning of terms easier

By applying semantic ontology analysis, we were able to find the groups of genes which belong to the same ontology cluster but which have a unique and specific GIT expression pattern. This kind of analysis allowed us to identify genes, which share similar functions in parallel regulatory networks. Additional file 3: Table S2 shows the ontology term combinations that were identified after applying semantic analysis on the GIT specific Ub-ligases and ubiquitination-related genes. Thus, ontology term combinations *GO:0018193: peptidyl-amino acid modification* and *GO:0042326: negative regulation of phosphorylation* unites five genes: Socs4, Socs5, Cbl, Socs1, Socs3 (Fig. 2A, B; Additional file 3: Table S2). Inside this ontology, genes Socs1, Socs3 are upregulated in the small intestine and downregulated in the colon, whereas genes Socs5 and Cbl exhibited the opposite expression. On the contrary, Socs4 does not show a significant difference in expression for colon and small intestine.

Another ontology term combination *GO:0045309: protein phosphorylated amino acid binding* and *GO:0044267: cellular protein metabolic process* includes Fbxw7, Nedd4, Btrc, Cblb and Socs3 genes (Fig. 2C, D; Additional file 3: Table S2). In this group, Socs3 is upregulated in the small intestine and downregulated in the colon, while genes Nedd4, Btrc, Cblb are characterized by the inversed expression pattern. Expression of Fbxw7 did not significantly differ between small intestine and colon. Interestingly, the same gene set (with additional Cbl gene, which is a paralog of Cblb) belongs to another ontology term combination *GO:0045309: protein phosphorylated amino acid binding* and *PW:0000417: ubiquitin, ubiquitin-like/proteasome degradation pathway*. For this ontology group, these genes display a similar tissue expression pattern (Fig. 2E, F; Additional file 3: Table S2) representing the theory that the same genes might be involved in multiple regulatory pathways.

Moreover, Socs genes represented the most illustrative expression pattern in the GIT, particularly Socs1, Socs3, Socs4, and Socs5. These genes appeared in 9 out of 10 ontology combinations. Thus, Socs5 was always downregulated in the small intestine and upregulated in the colon and stomach. Socs1 and Socs3 showed upregulation in the small intestine and downregulation in the colon and Socs1 was downregulated in the stomach tissue. Socs4 did not show any difference in expression between SI and colon which indicates its equal contribution for homeostasis of these tissues.

### Genes from the same ontology cluster alter their expression pattern after induced epithelial damage in colon

**Fig. 3:**
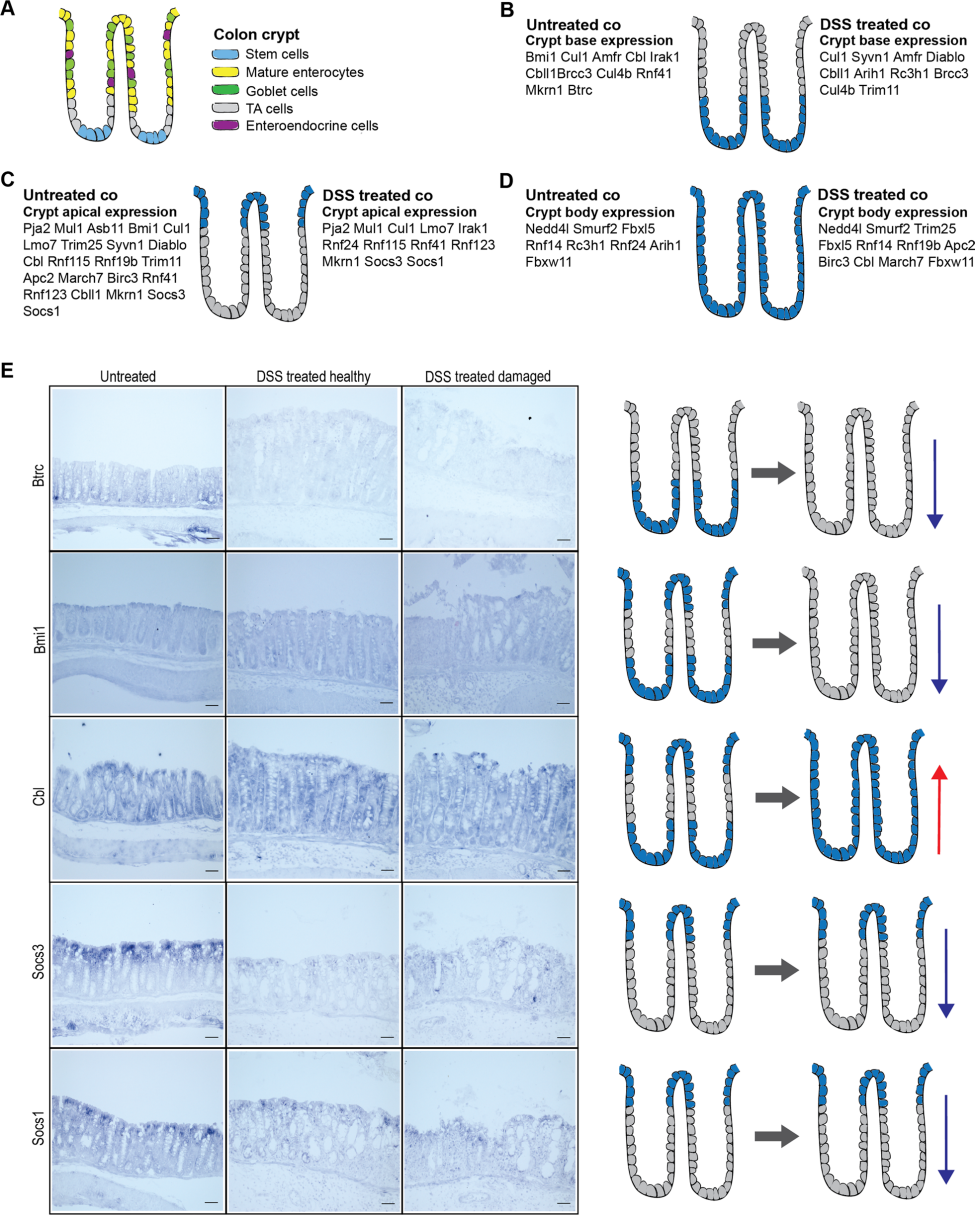
Spatial changes in expression of Ub-ligases of the same ontology group inside colonic crypts after induced epithelial damage. **a** Schematic illustration of the cell type residence in colonic crypt during homeostasis (based on (22, 38, 39, 41)). **b**–**d** Epithelial damage causes a shift in the expression pattern of ubiquitin ligases and ubiquitination-related genes in distal colon along the crypt axis. **e** In situ hybridization images of the DSS treated and untreated colon demonstrate spatial changes in expression of genes obtained from the ontology combination GO:0045309 and GO:0044267, GO:0045309 and PW:0000417. Red arrow depicts the gene Cbl expression upregulated after the DSS-induced damage and blue arrows display the other specific genes downregulated after the damage. Scale = 50 µm

In order to reveal possible parallel networks, we used a model of epithelial regeneration. We hypothesized that the genes involved in tissue regeneration might be masked by steady state homeostasis, thus their function might become apparent after tissue-challenged conditions, such as epithelial inflammatory damage. For this purpose, we induced epithelial damage by treating mice with DSS, which is widely used for mouse colitis models [23]. Following expression profiling detected 35 Ub-ligases and ubiquitination-related genes with altered expression in the distal colon after DSS treatment (Additional file 4; Additional file 5: Fig. S2). Then the localization of expression of 35 Ub-ligases and ubiquitination-related genes was monitored in the DSS treated and untreated distal colon tissue. It was observed that most of genes changed their expression pattern from the crypt base to apical part along the colon crypt, which is related to the disruption of homeostatic cell balance (Fig. 3A) in the crypt after damage. In the untreated colon, 22 out of 35 genes were detected on the luminal (apical) side of the crypt, whereas after DSS treatment only 12 genes remained, and others translocated either to the crypt base or spread over the entire crypt (Fig. 3B–D). This alteration in the expression pattern could be associated with the damaged and missing apical cells due to the inflammation. Similarly, a shift in expression localization was also observed in the case of genes that were originally expressed in the crypt base.

As for expression in the crypt body, we observed more genes that displayed expression in the affected area, some of which showed strong signal (Cbl, Fbxl5, Rnf19b, Apc2) (Fig. 3B–E; Additional file 5: Fig. S3). This observation might be a result of inflammation, local immune responses and/or robust epithelial regeneration that occurs after the DSS treatment, suggesting an essential role of those Ub-ligases in the mentioned processes. For instance, high presence of Apc2 (component of the anaphase-promoting complex/cyclosome (APC/C)) could be associated with dynamic cell cycle regulation for the maintaining of tissue homeostasis [33]. On the other hand, there were genes with significantly decreased expression after the treatment. Some E3s kept their original intramucosal location of expression (for example, Trim25, Smurf2, Trim11), whereas others were no longer detected (such as Bmi1, Asb11, March7, Btrc) (Additional file 5: Fig. S3) which indicates that these Ub-ligases are not necessary in the tissue damage response. Notably, together with Ub-ligases, Brcc3 (a part of multisubunit BRCC complex with Lys 63-linked deubiquitinating activity [34]) retained its expression pattern after the treatment, but expression levels were significantly decreased in the damaged tissue (Additional file 5: Fig. S3).

We further examined the response of genes that were grouped into the same ontology term combination GO:0045309 and GO:0044267, GO:0045309 and PW:0000417. In this analysis, Socs1 and Socs3 genes were downregulated in the colon while Btrc, Bmi1 and Cbl were upregulated. With the help of in situ hybridization we identified the specific expression regions in colon for each of these genes. Also, we saw that each expression region can differ under pathological conditions (Fig. 3E). In homeostasis, Btrc was highly expressed by the cells of the crypt apex, but its expression decreased remarkably after injury. A similar situation was observed for Bmi1, which was originally present in the apical part of the crypt and crypt base, respectively. Socs1 and Socs3 were localized to the crypt apex. In the damaged or regenerated tissue, they maintained this expression pattern, but their expression level decreased because of either missing or re-structured epithelia (Fig. 3E).

On the contrary, Cbl showed high expression at both the apical part of the crypt and crypt base, but DSS-induced damage significantly dispersed its expression through the entire crypt body. This could be explained by the potential communication of Cbl with signaling pathways maintaining stem/progenitor/mature cell balance during tissue regeneration (for example, through the protein tyrosine kinase-mediated signaling as mentioned in [35]. In conclusion, the results of ontological clustering and the subsequent in situ hybridization for the accordingly picked Ub-ligase genes displayed in Fig. 3 clearly suggest which cluster of Ub-ligases might be involved in colon mucosa inflammation and regeneration. This thorough analysis also exhibits the temporo-spatial dynamics of this Ub-ligase cluster function under these specific conditions.

### Ub-ligases from the same ontology cluster expressed by several cell types in colon indicating their compensatory potential

**Fig. 4:**
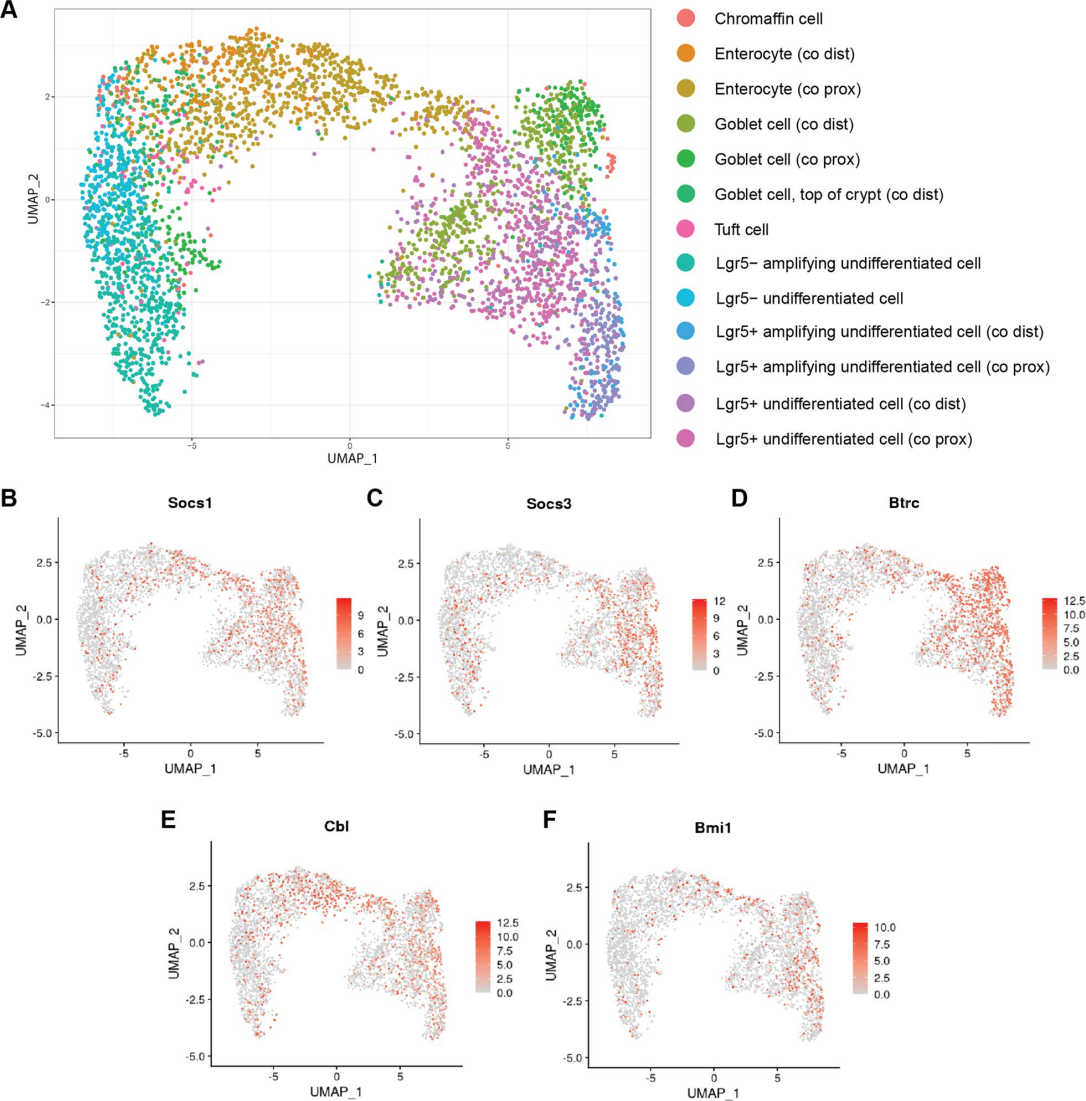
Distribution of Ub-ligases in colon. **a** UMAP analysis demonstrates that the colon Ub-ligases may be grouped into 13 cell specific clusters (labeled by colors). **b**–**f** Ub-ligases from the same ontology combination group displaying distributional expression among several cell types

To determine cell-type specific distribution of Ub-ligases in the colon, we used published single-cell RNA sequencing data of the murine colon as a reference [22]. We hypothesized that Ub-ligases from the same ontology cluster might be involved in the parallel signaling pathways in distinct cell types, but be active under the certain homeostatic condition or differentiation stage. This way, only Ub-ligase related genes were selected from the entire scRNA-seq dataset (n = 367) and were processed by the Seurat package [36]. For a cell subtype visualization, we performed principal component analysis (PCA), then the 10 most significant principal components were projected to two dimensions with Uniform Manifold Approximation and Projection technique (UMAP), the cells were then colored by their classification label [37]. We used established cell markers to determine cell types in proximal and distal colon, including enterocytes (Krt20+, Slc26a3+) [38], goblet cells (Atoh1+, Spdef+) [39, 40], tuft cells (Dclk1+) [41], chromaffin (also known as enteroendocrine) cells (Chga+, Chgb+) [42], proliferating (Lgr5-, Mki67+) and non-proliferating (Lgr5+ , Mki67−) stem cells (SCs) [17]. With the help of UMAP visualization, Ub-ligases were grouped into several clusters that correspond to specific cell type (Fig. 4A). However, there was no strict tissue association between distal and proximal parts of colon, and clusters there demonstrate just partial overlapping.

To further analyze the Ub-ligase distribution throughout the colon, we focused on genes that were clustered into the same ontology combination groups GO:0018193 and GO:0042326, GO:0045309 and GO:0044267, GO:0045309 and PW:0000417 (Additional file 3: Table S2). These genes showed a shift in expression after DSS-induced inflammation (Fig. 3E). Thus, Socs1 and Socs3 were mostly expressed by stem cells that clustered as Lgr5+ undifferentiated, Lgr5+ amplifying undifferentiated SCs and goblet cells both in proximal and distal colon, together with enterocyte cells of proximal colon (Fig. 4B, C). Besides this, Socs3 was also typical for Lgr5-undifferentiated SCs cluster (Fig. 4C). However, Socs1 and Socs3 genes were not typical either for chromaffin, tuft cells or goblet cell at the crypt apex. Btrc was abundantly expressed in the clusters of goblet cells, Lgr5+ amplifying undifferentiated and Lgr5+ undifferentiated SCs (Fig. 4D), and it was rather scarce in chromaffin, enterocyte, and tuft cell clusters. As for Cbl, Btrc displayed comparable distribution in all cell clusters with the higher concentration in the enterocyte and Lgr5+ undifferentiated SCs clusters (Fig. 4E). Finally, Bmi1 showed the lowest cell cluster specificity with equal distribution through all clusters with relatively higher specificity for Lgr5+ undifferentiated and Lgr5+ amplifying undifferentiated SCs clusters (Fig. 4F). These results support the evidence that Ub-ligases are expressed by various cell types along the tissue, but their functions slightly differ depending on the cell type, developmental stage and homeostatic condition, as it was observed also after the colon injury. This might bring a novel proof of semantically defined similarity of those Ub-ligases and compensatory potential in relation to each other.

## Discussion

To date, there have been many published reports on E3 Ub-ligases based on in vitro investigations. This provides valuable data regarding cellular physiology and homeostasis such as proliferation, cell growth, apoptosis, nucleic acids maintenance, metabolism, cell cycle etc., with either overexpressed or absent E3 Ub-ligases [1, 6, 43]. However, contextual information about their effect on a complex tissues, organs and organisms, including reciprocal regulations within a subpopulation of cells is missing in such models. Therefore, studying E3 Ub-ligases in vivo gives more information about the biological role of these enzymes and their implementation in the physiology of the entire organism. Yet, in vivo models are subjected to strong regulatory mechanisms relying on compensatory effects of alternative pathways.

The ability of a biological system to maintain homeostasis in the presence of mutations is determined by the term genetic robustness. This feature is evolutionarily essential for the organism’s survival in the case of gene dysfunction and can be achieved via regulatory pathway intercommunication [44, 45]. However, this could cause difficulties to analyze the animal models, when gene targeting does not lead to the expected abundant or severe phenotype. After first being reported in *Drosophila* as transcriptional dosage compensation of the X chromosome [46], genetic robustness was then described in many model organisms from yeast [47] to mammals [48]. To explain the genetic robustness phenomenon, researchers proposed several mechanisms, such as functional redundancy of homologous genes [49], adaptive mutations [50], rewriting of genetic networks [51], genetic compensation, and transcriptional adaptation [44].

To gain a deeper understanding of genetic and functional compensation, we propose the use of Semantic clustering analysis [11, 12] to statistically predict and describe semantically coherent gene bi-clusters in the context of functional gene classification for specific cell type in the tissue. To test a model of semantic clustering analysis, we compared expression of E3 ubiquitin ligases and ubiquitination-related genes in three main segments of the gastrointestinal tract, i.e. in stomach, small intestine and colon. As a first outcome, the small intestine appears to possess all the ligases expressed at the lowest level. Knowing this we used the expression in small intestine as a reference level for stomach and colon for the ontology analyses, dividing expressed genes according to their function in cells and tissues. These analyses revealed that the small intestine is characterized by genes involved in the maintenance of the immune system, and that genes playing roles in the catabolic processes are typical for the colon.

It has been discussed if compensatory activity of redundant genes may or may not correlate with their similarities in sequence or structure and in common origin [52]. These facts complicate compensatory pathway identification. Applying the theory above, we were able to reveal ten groups of Ub-ligases that share the same ontologies, but that carry the GIT specific expression pattern. Notably, the genes from the same ontology combination group have not described as redundant before, which gives an interesting hint for a detailed study of those genes in signaling pathway networks.

In order to test the possible identified parallel networks in a biological system, we used a mouse model of epithelial regeneration. We hypothesized that genes involved in tissue regeneration might be masked by steady state homeostasis, but they may expose their functions after changes in tissue function under challenged conditions. Therefore, we induced epithelial damage by treating mice with DSS. We observed that epithelial damage in the colon activated intracellular signaling transduction with the activation of particular genes functions that differ from their normal role in homeostasis. This suggestion was also supported by our approach to classify Ub-ligases based on their cell specificity. We did not observe any strict cell specificity and the tested Ub-ligases were found to be present in various cell types. This observation refers to the ability of Ub-ligases to participate in the regulation of several signaling pathways in specific clusters. Yet, such regulation can be significantly different depending on tissue type, developmental stage and homeostatic condition.

Taken together, the semantic clustering analysis of GIT specific Ub-ligases and ubiquitination-related genes allows the ability to statistically define compensatory gene clusters consisting of the same genes involved in the distinct regulatory pathways vs a few different genes playing roles in functionally similar signaling pathways. Such an approach could find potential application, for instance, in cancer therapy development as redundancy/substitution of certain genes has also been described during cancerogenesis. In this case, redundant genes cover the potential harmful effects of mutation, and cancer progression depends on the effective functional setup between defective genes and their compensatory partners [52]. The most illustrative expression pattern in GIT semantic ontologies combinations showed members of the Socs family. Besides their role in immune response regulation as suppressors of cytokine signaling, some members of the Socs family were described to participate in tumor progression [53]. For instance, SOCS1 downregulation was described in hepatocellular carcinoma [54], cervical [55], ovarian and breast cancer [56]. Aberrant expression of SOCS1 and SOCS3 has been described in human colorectal cancer, where SOCS3 overexpression inhibits proliferation, migration and invasiveness of tumor cells [57], while SOCS1 overexpression has pro-oncogenic activity [58]. In this manner, it would be meaningful to further study Socs genes together with other genes from the same ontology group in terms of compensatory potential during cancerogenesis and other GIT disease progression.

Having obtained an overview of Ub-ligases clustering, it would be interesting to apply the semantic clustering approach for studying the redundancy of these enzymes within their families, such as proteases, phosphatases, kinases etc. Their important biological roles indirectly suggest their high compensatory potential. Operating with the knowledge of ontology relationship among genes will help to choose the relevant animal model for study of a particular disease and future therapy development.

## Conclusion

The aim of this study was to explore gastrointestinal tract specific Ub-ligases and ubiquitination-related genes, define their dominant biological roles in homeostasis and possible contribution to alternative compensatory networks. By applying an improved ontology-based clustering method, i.e. semantic clustering, we performed Ub-ligase profiling and revealed ten ontology combination Ub-ligases groups that potentially exhibit redundant features in GIT. Testing the identified compensatory networks in a biological system showed that genes from the same ontology cluster simultaneously alter their expression pattern after induced epithelial damage, exposing their compensatory activity during tissue regeneration.

## Supplementary Information

**Additional file 1.** Expression profiling of Ub-ligases and ubiquitination-related genes in stomach, small intestine and colon of WT mice.

**Additional file 2.** Extended version of Table 1. Significantly different ontologies for different tissue.

**Additional file 3: Table S1.** Primers for RNA probe preparation. **Table S2.** Selection of ontology combinations of genes, which are differently expressed in various parts of GIT.

**Additional file 4.** Expression profiling of Ub-ligases and ubiquitination-related genes in colon of DSS treated and control mice.

**Additional file 5.** **Fig. S1.** Workflow of semantic cluster analysis. The original qPCR data matrix is divided into 3 groups of samples according to their type of tissue. For each tissue combination, we performed statistical analysis to reveal two sets of genes: organ specific and general genes. Together with ontologies, the sem1R algorithm is run for each set of genes individually to induce a hypothesis describing specifics of organ specific genes over the general ones. **Fig. S2.** Heatmap of the differential expression of Ub-ligases and ubiquitination-related genes after induced epithelial damage. **Fig. S3.** Differential expression of Ub-ligases and ubiquitination-related genes after induced epithelial damage.

## Data Availability

All data generated or analyzed during this study are included in this published article and/or supplementary file.

## Abbreviations

CO: Colon, large intestine
DSS: Dextran sodium sulphate
GIT: Gastrointestinal tract
GSEA: Gene set enrichment analysis
PCA: Principal component analysis
SI: Small intestine
sc-RNA-seq: Single-cell RNA sequencing
SC: Stem cell
Ub: Ubiquitin
UMAP: Uniform manifold approximation and projection technique
