# Supplementary material for: Semantic clustering analysis of E3-ubiquitin ligases in gastrointestinal tract defines genes ontology clusters with tissue expression patterns
Source: BMC Gastroenterol. 2022 Apr 12;22:186. doi: 10.1186/s12876-022-02265-2 (PMC9006408; doi:10.1186/s12876-022-02265-2)
Supplement: Supplementary file 3 — Additional file 3: Table S1. Primers for RNA probe preparation. Table S2. Selection of ontology combinations of genes, which are differently expressed in various parts of GIT. [file 12876_2022_2265_MOESM3_ESM.docx]

**Table S1.** Primers for RNA probe preparation

| **Gene** | **Primer pair sequence (5'->3')** |
| --- | --- |
| Bmi1 | F: CGCTTGGCTCGCATTCATTTT  R: AAGAGGTGGAGGGAACACC |
| Fbxl5 | F: GCTCTCCGAGATGCTGAGTC  R: GCCTTGCCTGCACTTTTCAG |
| Fbxw11 | F: GCCCCTCCCAGTGAATGAA  R: TGAGAAATGTGACAGAACAGAGG |
| Neddl4 | F: CGCTGAACCCAAAGTGGAATG  R: GATTCCTCACCACCCGTGAC |
| March1 | F: GGCCTACAACCGTGTGATC  R: GTTGCCTTTGTAGCTCGTGT |
| March2 | F: CGGAACAGGTTTCTGGGACCAT  R: GTAGCCAGCAAGGAGTGATGT |
| March5 | F: TGCGCTAGTGGAGACTTGTG  R: TCAAGAGCTCCCTTGGGTTC |
| March6 | F: GCCATTTCTCCCTTGTTGGC  R: TGGTAAAGTAAGGCAAATGAGGC |
| March7 | F: CGGTGGAAGAGGGAAACCTT  R: CAACCCCCATCTTCGCCTAA |
| March8 | F: GTCTGCCTTCTCTCGCACTT  R: CAGACCACAGAGAAGCCTGG |
| Cul1 | F: TGGAGTCCCTCCTTCCAAGTC  R: GTCACAGTACCGAGCAAGCAG |
| Cul2 | F: GAGCGAGCAACATGGAATGACC  R: GTAAGGTTACTGGTCGCTCTGAGG |
| Cul3 | F: TGATTGCCAGAGAGCGGAAAG  R: ACTTTGGTGTGGCTGACTGAG |
| Cul4A | F: AACGAAGTGGAGAGGCTGTG  R: TTCCATGGGTGTGTACGTGG |
| Cul4B | F: TGCTACCCTCCATTTGGGAC  R: CGGCTCCACATTCGTGTTTC |
| Cul5 | F: CGTCTCCTCAAGCGTTTGCATG  R: GGTCACCAGCGCATTTACACAG |
| Hsp90 ab | F: TTTATCCGCGGTGTGGTTGA  R: ATCATGCGGTAGATGCGGTT |
| Btrc | F: GGCCTTCGAGACAACACCAT  R: ACATTAACGAGTCCTGGGCAA |
| Socs1 | F: AGTAGGATGGTAGCACGCAA  R: ACATGGAGAGGTAGGAGTGGAA |
| Socs3 | F: CGAGGCGGCTCTAACTCTGA  R: ACGTTGGAGGAGAGAGGTCG |

**Table S2.** Selection of ontology combinations of genes which are differently expressed in various parts of GIT.

| **Comparison** | **Gene** | **Ontology** |
| --- | --- | --- |
| small intestine - colon | Fbxw7 | ID: GO:0018193: peptidyl-amino acid modification DEF: "The alteration of an amino acid residue in a peptide." [GOC:mah] level: 6  ID: GO:0030154: cell differentiation DEF: "The process in which relatively unspecialized cells, e.g. embryonic or regenerative cells, acquire specialized structural and/or functional features that characterize the cells, tissues, or organs of the mature organism or some other relatively stable phase of the organism's life history. Differentiation includes the processes involved in commitment of a cell to a specific fate and its subsequent development to the mature state." [ISBN:0198506732] level: 3 |
|  | Mul1 |  |
|  | Trim28 |  |
|  | Socs5↓ |  |
|  | Neurl1a↓ |  |
|  | Atrx↓ |  |
|  | Pias2↓ |  |
|  | Vprbp↓ |  |
|  | Pias1↓ |  |
|  | Bmi1↓ |  |
|  | Socs1↑ |  |
|  | Socs3↑ |  |
| small intensive - colon | Socs4 | ID: GO:0018193: peptidyl-amino acid modification DEF: "The alteration of an amino acid residue in a peptide." [GOC:mah] level: 6  ID: GO:0042326: negative regulation of phosphorylation DEF: "Any process that stops, prevents or decreases the rate of addition of phosphate groups to a molecule." [GOC:jl] level: 6 |
|  | Socs5↓ |  |
|  | Cbl↓ |  |
|  | Socs1↑ |  |
|  | Socs3↑ |  |
| small intestine - colon | Fbxw7 | ID: GO:0018193: peptidyl-amino acid modification DEF: "The alteration of an amino acid residue in a peptide." [GOC:mah] level: 6  ID: GO:0048869: cellular developmental process DEF: "A biological process whose specific outcome is the progression of a cell over time from an initial condition to a later condition." [GOC:isa_complete] level: 2 |
|  | Mul1 |  |
|  | Trim28 |  |
|  | Socs5↓ |  |
|  | Neurl1a↓ |  |
|  | Atrx↓ |  |
|  | Pias2↓ |  |
|  | Vprbp↓ |  |
|  | Pias1↓ |  |
|  | Bmi1↓ |  |
|  | Socs1↑ |  |
|  | Socs3↑ |  |
| small intestine - colon | Rnf138 | ID: GO:0019901: protein kinase binding DEF: "Interacting selectively and non-covalently with a protein kinase, any enzyme that catalyzes the transfer of a phosphate group, usually from ATP, to a protein substrate." [GOC:jl] level: 5  ID: GO:0006464: cellular protein modification process DEF: "The covalent alteration of one or more amino acids occurring in proteins, peptides and nascent polypeptides (co-translational, post-translational modifications) occurring at the level of an individual cell. Includes the modification of charged tRNAs that are destined to occur in a protein (pre-translation modification)." [GOC:go_curators] level: 5 |
|  | Rnf41 |  |
|  | Traf2 |  |
|  | Socs5↓ |  |
|  | Fbxw5↓ |  |
|  | Pja2↓ |  |
|  | Cbl↓ |  |
|  | Fbxo5↓ |  |
|  | Fbxo7↓ |  |
|  | Socs1↑ |  |
| small intestine - colon | Rnf138 | ID: GO:0019901: protein kinase binding DEF: "Interacting selectively and non-covalently with a protein kinase, any enzyme that catalyzes the transfer of a phosphate group, usually from ATP, to a protein substrate." [GOC:jl] level: 5  ID: GO:0044267: cellular protein metabolic process DEF: "The chemical reactions and pathways involving a specific protein, rather than of proteins in general, occurring at the level of an individual cell. Includes cellular protein modification." [GOC:jl] level: 4 |
|  | Rnf41 |  |
|  | Traf2 |  |
|  | Socs5↓ |  |
|  | Fbxw5↓ |  |
|  | Pja2↓ |  |
|  | Cbl↓ |  |
|  | Fbxo5↓ |  |
|  | Fbxo7↓ |  |
|  | Socs1↑ |  |
| small intension - colon | Rnf138 | ID: GO:0019901: protein kinase binding DEF: "Interacting selectively and non-covalently with a protein kinase, any enzyme that catalyzes the transfer of a phosphate group, usually from ATP, to a protein substrate." [GOC:jl] level: 5  ID: GO:0070647: protein modification by small protein conjugation or removal DEF: "A protein modification process in which one or more groups of a small protein, such as ubiquitin or a ubiquitin-like protein, are covalently attached to or removed from a target protein." [GOC:mah] level: 6 |
|  | Rnf41 |  |
|  | Traf2 |  |
|  | Socs5↓ |  |
|  | Fbxw5↓ |  |
|  | Pja2↓ |  |
|  | Cbl↓ |  |
|  | Fbxo5↓ |  |
|  | Fbxo7↓ |  |
|  | Socs1↑ |  |
| small intestine - colon | Socs4 | ID: GO:0042326: negative regulation of phosphorylation DEF: "Any process that stops, prevents or decreases the rate of addition of phosphate groups to a molecule." [GOC:jl] level: 6  ID: GO:0018193: peptidyl-amino acid modification DEF: "The alteration of an amino acid residue in a peptide." [GOC:mah] level: 6 |
|  | Socs5↓ |  |
|  | Cbl↓ |  |
|  | Socs1↑ |  |
|  | Socs3↑ |  |
| small intestine - colon | Fbxw7 | ID: GO:0045309: protein phosphorylated amino acid binding DEF: "Interacting selectively and non-covalently with a phosphorylated amino acid residue within a protein." [GOC:go_curators] level: 4  ID: GO:0044267: cellular protein metabolic process DEF: "The chemical reactions and pathways involving a specific protein, rather than of proteins in general, occurring at the level of an individual cell. Includes cellular protein modification." [GOC:jl] level: 4 |
|  | Nedd4↓ |  |
|  | Btrc↓ |  |
|  | Cblb↓ |  |
|  | Socs3↑ |  |
| small intestine - colon | Fbxw7 | ID: GO:0045309: protein phosphorylated amino acid binding DEF: "Interacting selectively and non-covalently with a phosphorylated amino acid residue within a protein." [GOC:go_curators]  level: 4  ID: PW:0000417: ubiquitin, ubiquitin-like/proteasome degradation pathway DEF: "The pathway for the ATP-dependent non-lysosomal proteolysis catalyzed by the 26S proteasome and for which ubiquitination, the post-translational covalent conjugation of ubiquitin, or ubiquitin-like, to target proteins is a key signal." [PMID:10993052] level: 4 |
|  | Nedd4↓ |  |
|  | Btrc↓ |  |
|  | Cbl↓ |  |
|  | Cblb↓ |  |
|  | Socs3↑ |  |
| small intestine - colon | Rnf41 | ID: GO:0045619: regulation of lymphocyte differentiation DEF: "Any process that modulates the frequency, rate or extent of lymphocyte differentiation." [GOC:go_curators] level: 5  ID: GO:0036211: protein modification process DEF: "The covalent alteration of one or more amino acids occurring in proteins, peptides and nascent polypeptides (co-translational, post-translational modifications). Includes the modification of charged tRNAs that are destined to occur in a protein (pre-translation modification)." [GOC:bf, GOC:jl] level: 4 |
|  | Socs5↓ |  |
|  | Fbxo7↓ |  |
|  | Bmi1↓ |  |
|  | Socs1↑ |  |
|  | Trim28 |  |
|  | Pias1↑ |  |
|  | Pias2↑ |  |
|  | Pcgf2↑ |  |
|  | Bcor↑ |  |
|  | Atrx↑ |  |
| stomach - small intestine | Fbxw8 | ID: GO:0010975: regulation of neuron projection development DEF: "Any process that modulates the rate, frequency or extent of neuron projection development. Neuron projection development is the process whose specific outcome is the progression of a neuron projection over time, from its formation to the mature structure. A neuron projection is any process extending from a neural cell, such as axons or dendrites (collectively called neurites)." [GOC:dph, GOC:tb] level: 6  ID: GO:0004842: ubiquitin-protein transferase activity DEF: "Catalysis of the transfer of ubiquitin from one protein to another via the reaction X-Ub + Y --> Y-Ub + X, where both X-Ub and Y-Ub are covalent linkages." [GOC:BioGRID, GOC:jh2, PMID:9635407] level: 4 |
|  | Rnf6 |  |
|  | Mul1↓ |  |
|  | Fbxo7↑ |  |
|  | Neurl1a↑ |  |
|  | Nedd4↑ |  |
|  | Park2↑ |  |
| stomach - small intestine | Topors | ID: GO:0043005: neuron projection DEF: "A prolongation or process extending from a nerve cell, e.g. an axon or dendrite." [GOC:jl, http://www.cogsci.princeton.edu/~wn/] level: 3  ID: GO:0044444: cytoplasmic part DEF: "Any constituent part of the cytoplasm, all of the contents of a cell excluding the plasma membrane and nucleus, but including other subcellular structures." [GOC:jl] level: 3 |
|  | Trim9 |  |
|  | Mul1↓ |  |
|  | Fbxo7↑ |  |
|  | Fbxo2↑ |  |
|  | Neurl1a↑ |  |
|  | Nedd4↑ |  |
|  | Trim3↑ |  |
|  | Park2↑ |  |
| stomach - small intestine | Pax6 | ID: GO:0043412: macromolecule modification DEF: "The covalent alteration of one or more monomeric units in a polypeptide, polynucleotide, polysaccharide, or other biological macromolecule, resulting in a change in its properties." [GOC:go_curators] level: 4  ID: GO:0051246: regulation of protein metabolic process DEF: "Any process that modulates the frequency, rate or extent of the chemical reactions and pathways involving a protein." [GOC:ai] level: 4  ID: GO:0007399: nervous system development DEF: "The process whose specific outcome is the progression of nervous tissue over time, from its formation to its mature state." [GOC:dgh] level: 4 |
|  | Mnat1 |  |
|  | Cdc20 |  |
|  | Trim71 |  |
|  | Trim32 |  |
|  | Traf6 |  |
|  | Mul1↓ |  |
|  | Fbxo7↑ |  |
|  | Mylip↑ |  |
|  | Bmi1↑ |  |
|  | Atrx↑ |  |
|  | Enc1↑ |  |
|  | Neurl1a↑ |  |
|  | Nedd4↑ |  |
|  | Park2↑ |  |
| stomach - small intestine | Trim32 | ID: GO:0045597: positive regulation of cell differentiation DEF: "Any process that activates or increases the frequency, rate or extent of cell differentiation." [GOC:go_curators] level: 3  ID: GO:0051247: positive regulation of protein metabolic process DEF: "Any process that activates or increases the frequency, rate or extent of the chemical reactions and pathways involving a protein." [GOC:ai] level: 4 |
|  | Socs1↓ |  |
|  | Mul1↓ |  |
|  | Bmi1↑ |  |
|  | Neurl1a↑ |  |
|  | Socs5↑ |  |
|  | Park2↑ |  |
| stomach - small intestine | Rnf41 | ID: GO:0045619: regulation of lymphocyte differentiation DEF: "Any process that modulates the frequency, rate or extent of lymphocyte differentiation." [GOC:go_curators] level: 5  ID: GO:0031399: regulation of protein modification process DEF: "Any process that modulates the frequency, rate or extent of the covalent alteration of one or more amino acid residues within a protein." [GOC:mah, GOC:tb] level: 5 |
|  | Socs1↓ |  |
|  | Fbxo7↑ |  |
|  | Bmi1↑ |  |
|  | Socs5↑ |  |
|  | Whsc1 |  |
|  | Pias1↑ |  |
|  | Pias2↑ |  |
|  | Pcgf2↑ |  |
|  | Bcor↑ |  |
|  | Atrx↑ |  |
| stomach - small intestine | Pias4 | ID: PW:0000209: Jak-Stat signaling pathway DEF: "The Jak-Stat pathway is a main intracellular cascade initiated primarily in response to cytokine and also other ligand signaling. Four Janus kinases (Jak) and seven signal transducers and activators of transcription (Stat) families of proteins mediate the action of almost 40 cytokine receptors, including the receptor for leptin. Combinations between four Jak(s) and seven Stat(s) shape the outcome of ligand triggered signaling through the various receptors." [GO:0007259, KEGG:map04630, MCW library:Handbook of cellular and molecular immunology, PMID:12039028, PMID:14668806, PMID:14737178, PMID:17312100] level: 3  ID: GO:0019899: enzyme binding DEF: "Interacting selectively and non-covalently with any enzyme." [GOC:jl]  level: 3 |
|  | Socs1↓ |  |
|  | Pias1↑ |  |
|  | Pias2↑ |  |
|  | Socs5↑ |  |

Arrow code depicts upregulation (↑) or downregulation (↓) in the tissue. Genes without any significant changes in expression are not labeled with an arrow.
