# Supplementary material for: Semantic clustering analysis of E3-ubiquitin ligases in gastrointestinal tract defines genes ontology clusters with tissue expression patterns
Source: BMC Gastroenterol. 2022 Apr 12;22:186. doi: 10.1186/s12876-022-02265-2 (PMC9006408; doi:10.1186/s12876-022-02265-2)
Supplement: Supplementary file 5 — Additional file 5. Fig. S1. Workflow of semantic cluster analysis. The original qPCR data matrix is divided into 3 groups of samples according to their type of tissue. For each tissue combination, we performed statistical analysis to reveal two sets of genes: organ specific and general genes. Together with ontologies, the sem1R algorithm is run for each set of genes individually to induce a hypothesis describing specifics of organ specific genes over the general ones. Fig. S2. Heatmap of the differential expression of Ub-ligases and ubiquitination-related genes after induced epithelial damage. Fig. S3. Differential expression of Ub-ligases and ubiquitination-related genes after induced epithelial damage. [file 12876_2022_2265_MOESM5_ESM.pdf]

Fig. S1 Workflow of semantic cluster analysis. The original qPCR data matrix is divided into 3 groups of samples according to their type of tissue. For each tissue combination, we performed statistical analysis to reveal two sets of genes: organ specific and general genes. Together with ontologies, the sem1R algorithm is run for each set of genes individually to induce a hypothesis describing specifics of organ specific genes over the general ones.

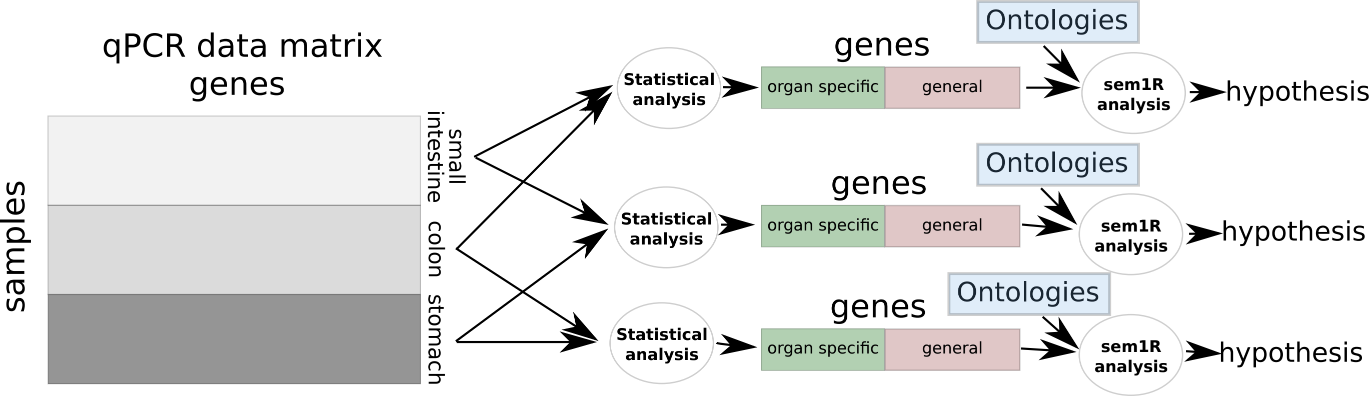
qPCR data matrix
genes
samples
small intestine
colon
stomach
Statistical analysis
Statistical analysis
Statistical analysis
genes
organ specific
general
genes
organ specific
general
genes
organ specific
general
Ontologies
Ontologies
Ontologies
sem1R analysis
sem1R analysis
sem1R analysis
hypothesis
hypothesis
hypothesis

Fig. S2 Heatmap of the differential expression of Ub-ligases and ubiquitination-related genes after induced epithelial damage.

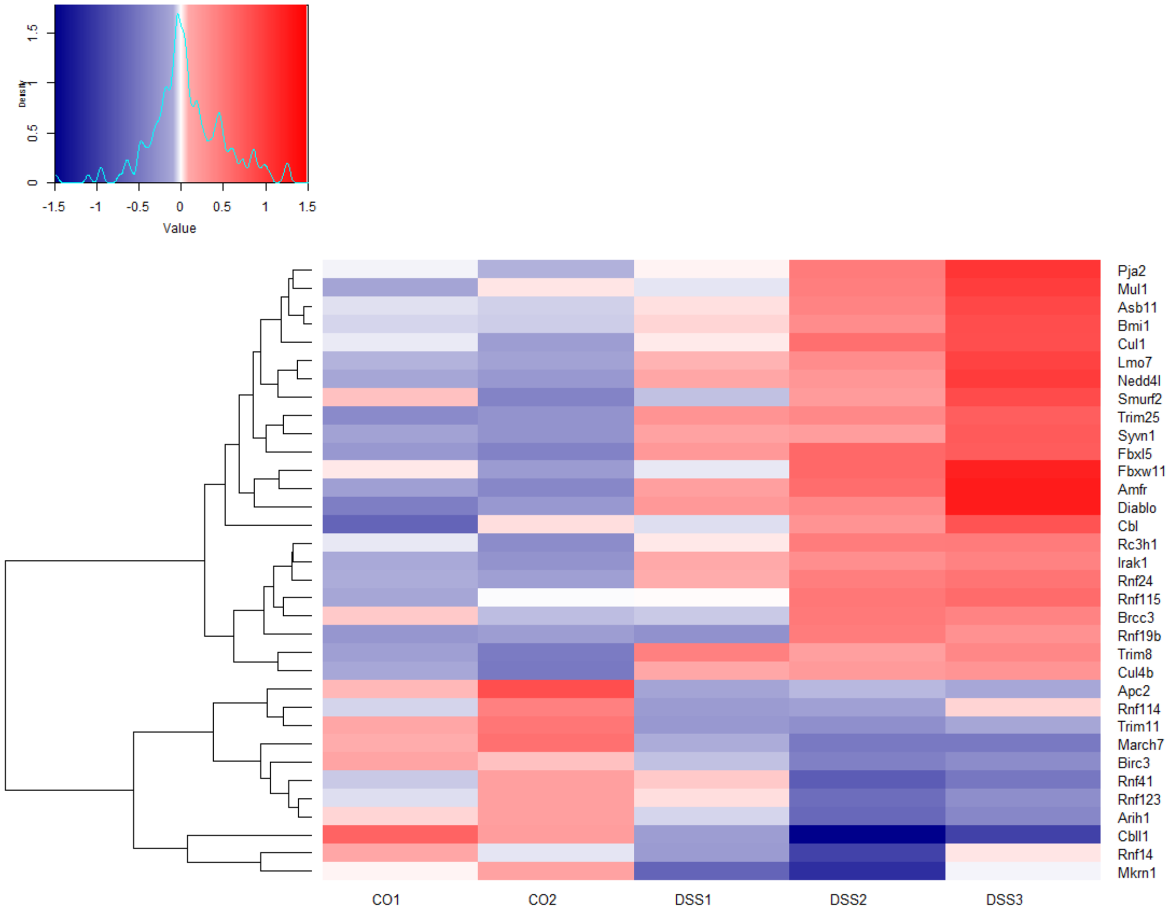

Fig. S3 Differential expression of Ub-ligases and ubiquitination-related genes after induced epithelial damage.

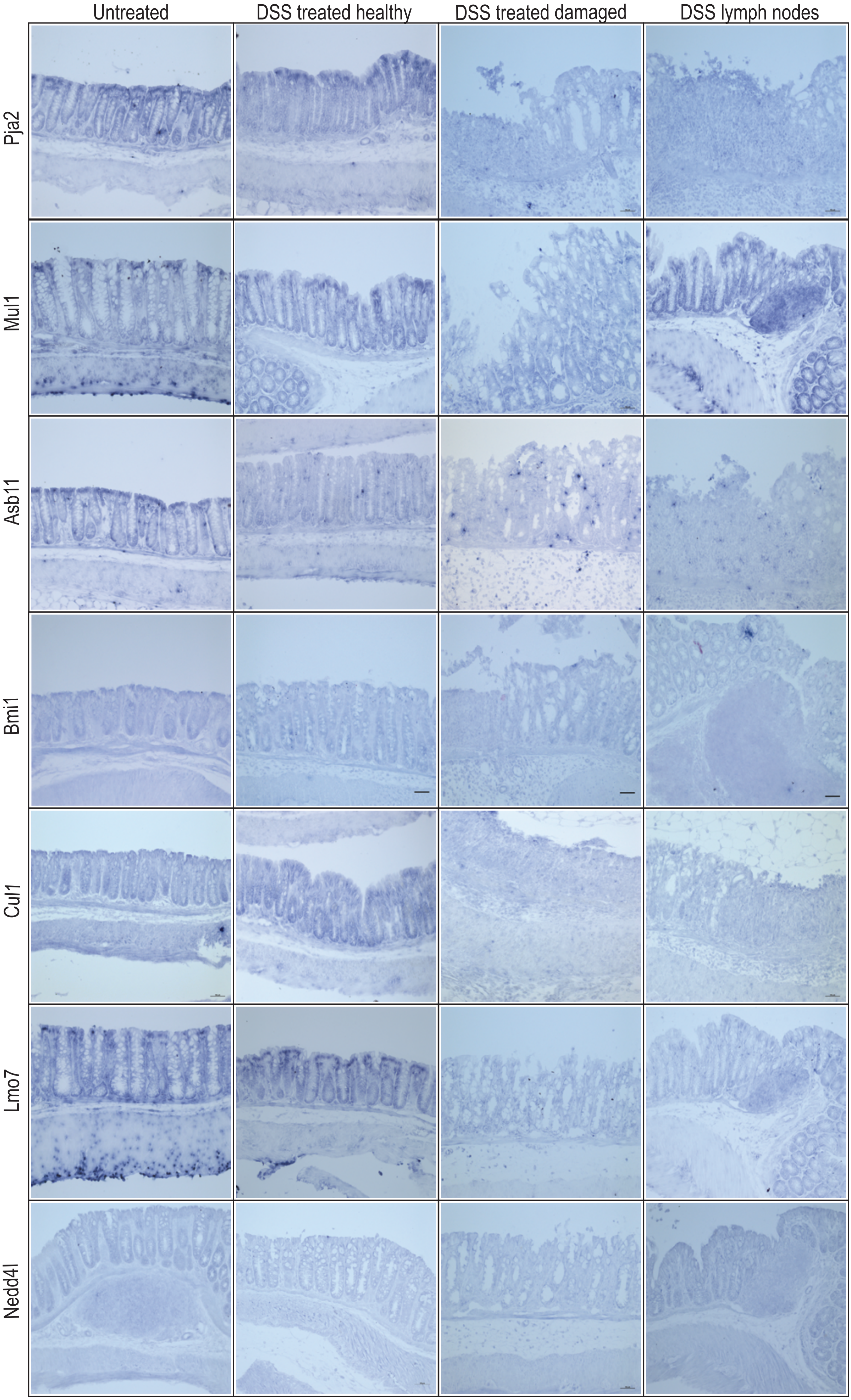
Untreated
DSS treated healthy
DSS treated damaged
DSS lymph nodes
Pja2
Mul1
Asb11
Bmi1
Cul1
Lmo7
Nedd4l

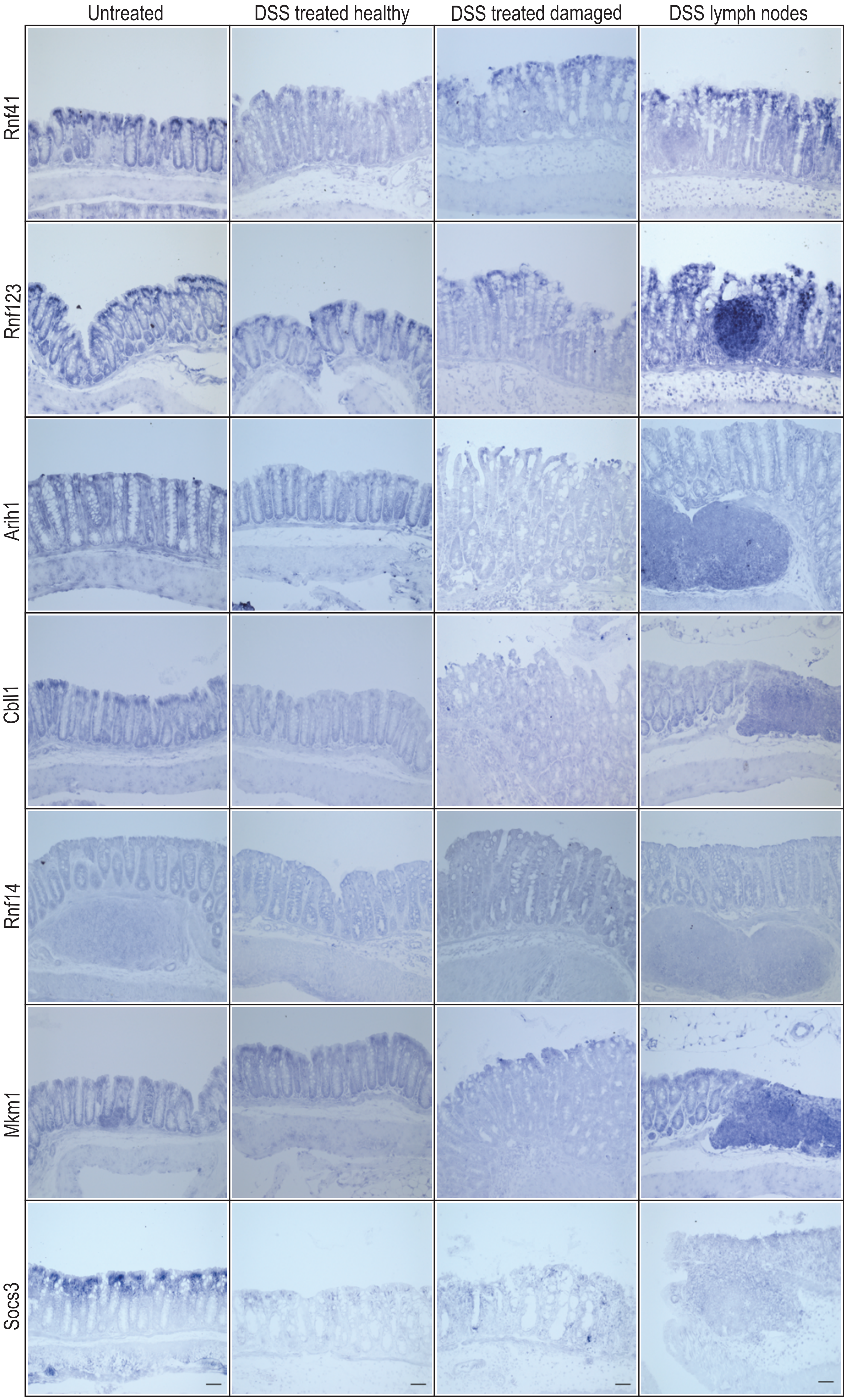

Untreated
DSS treated healthy
DSS treated damaged
DSS lymph nodes
Rnf41
Rnf123
Arih1
Cbll1
Rnf14
Mkm1
Socs3

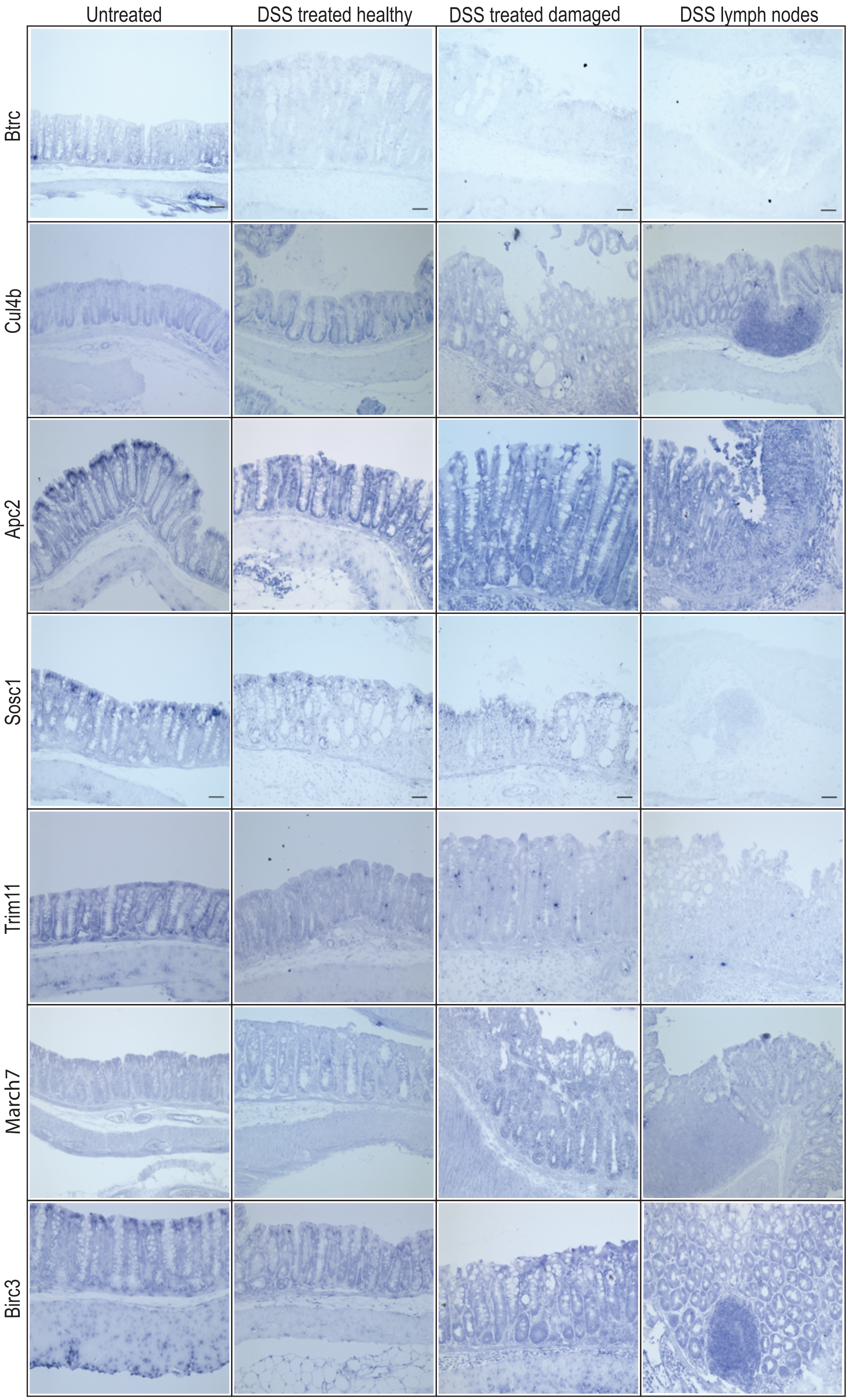

Untreated
DSS treated healthy
DSS treated damaged
DSS lymph nodes
Btrc
Cul4b
Apc2
Sosc1
Trim11
March7
Birc3

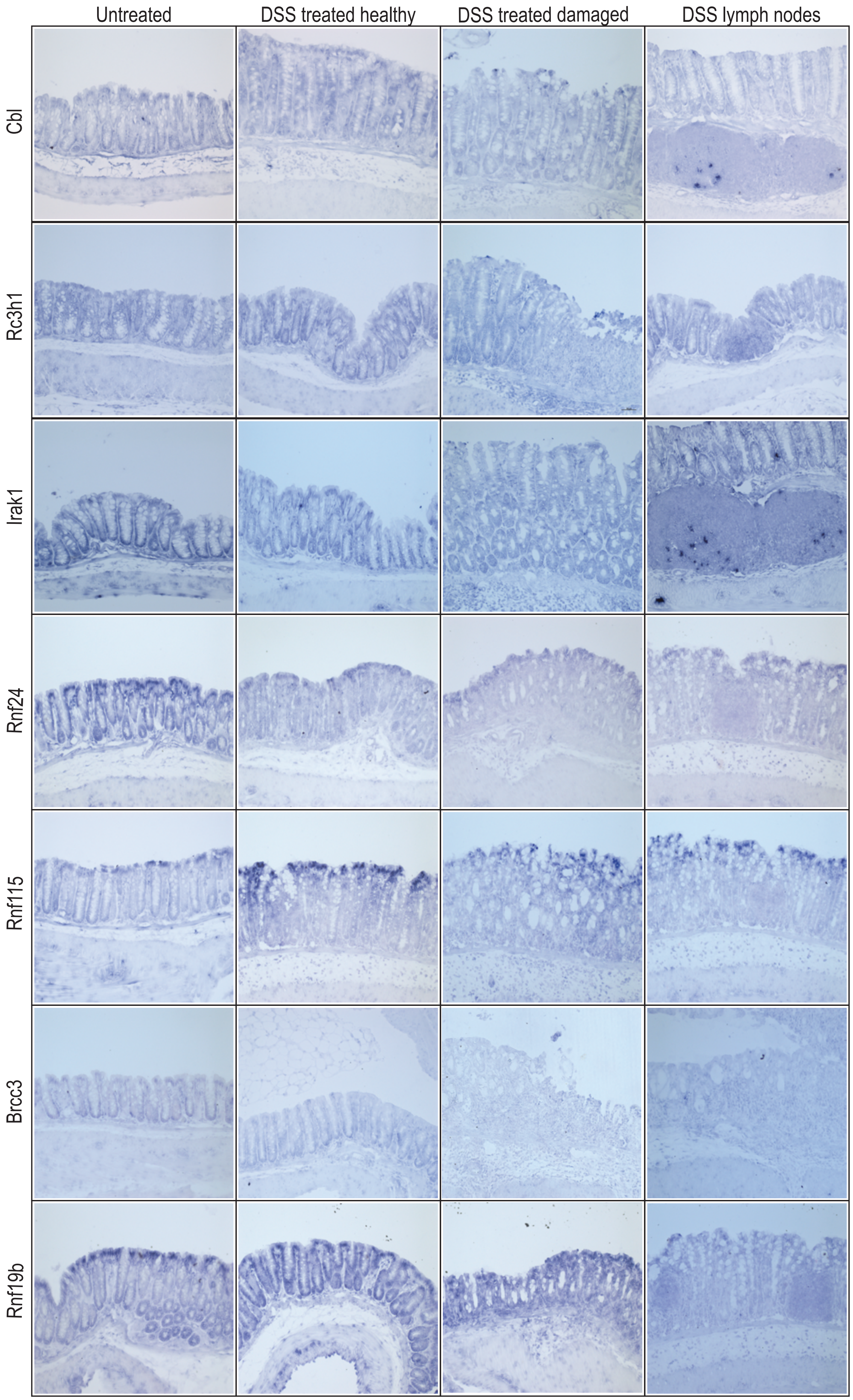
Untreated
DSS treated healthy
DSS treated damaged
DSS lymph nodes
Cbl
Rc3h1
Irak1
Rnf24
Rnf115
Brcc3
Rnf19b

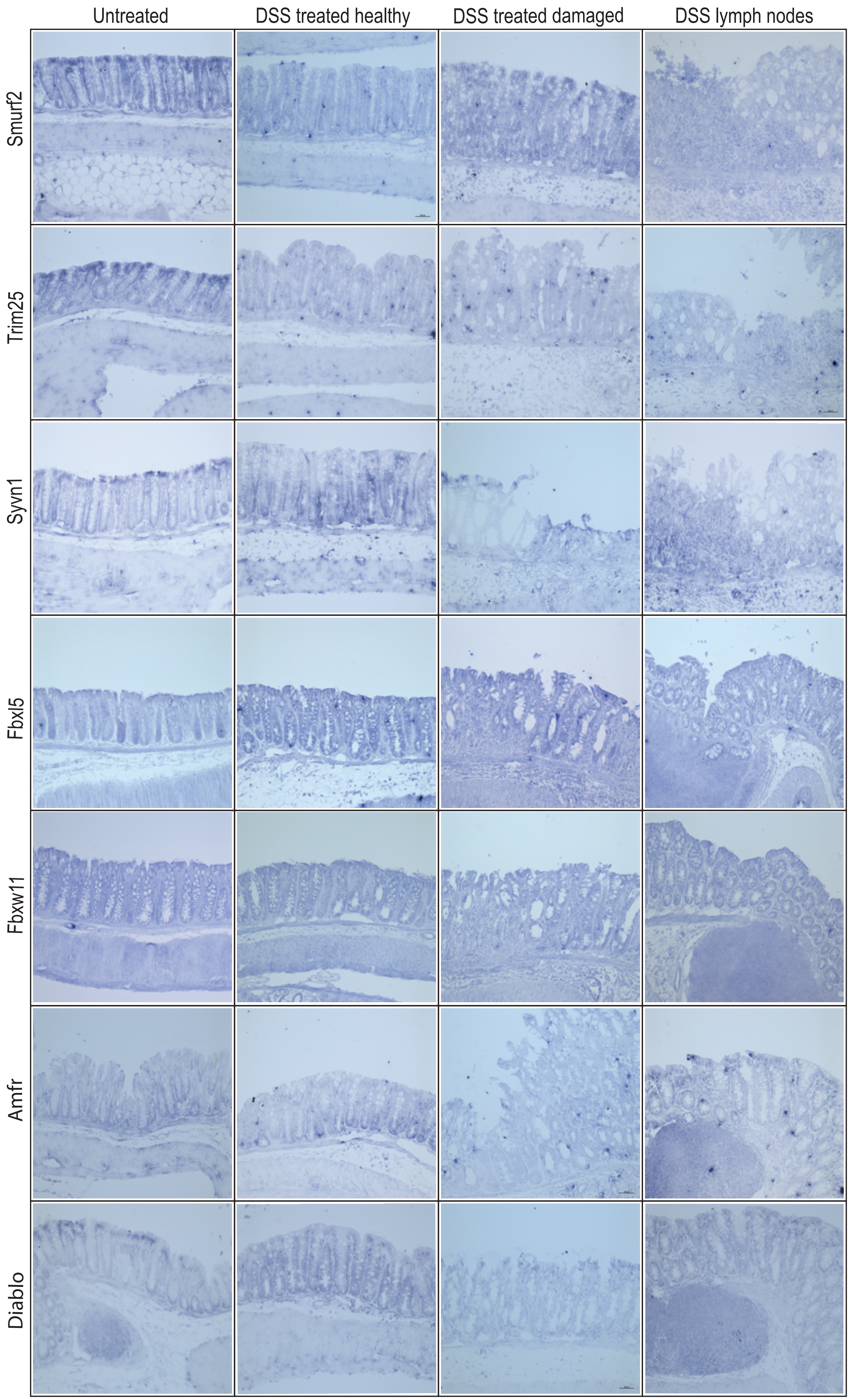

Untreated
DSS treated healthy
DSS treated damaged
DSS lymph nodes
Smurf2
Trim25
Syvn1
Fbxl5
Fbxw11
Amfr
Diablo
